# The General Hospital in Vienna

**Published:** 1878-05

**Authors:** 


THE GENERAL HOSPITAL IN VIENNA.—A new bathroom, which contains seven continuous water-beds or baths, has recently been added to this hospital. The continuous water-bath has proved exceedingly valuable in the treatment of certain diseases of the skin, and still more of desperate surgical cases, such as severe burns, and phagedenic ulcers, etc. The patients find themselves very comfortable in the bath, which usually relieves pain rapidly even when severe. The baths are so arranged that the patients can sleep comfortably, and move about in them without difficulty—a very essential point when the sufferers are obliged to remain constantly in them for months at a time. The additional facilities afforded by the new bath-room for the administration of these baths, are a valuable addition to the therapeutic resources of the hospital, and deserve the careful consideration of all hospital authorities and practical surgeons.—*Medical Record.*

